# Além do Paradigma IAMCSST-IAMSSST: Proposta do Instituto Dante Pazzanese para o Diagnóstico de Oclusão Coronariana Aguda

**DOI:** 10.36660/abc.20230733

**Published:** 2024-06-21

**Authors:** José Nunes De Alencar, Fausto Feres, Mariana Fuziy Nogueira De Marchi, Kleber Gomes Franchini, Matheus Kiszka Scheffer, Sandro Pinelli Felicioni, Ana Carolina Muniz Costa, Rinaldo Carvalho Fernandes, Hugo Ribeiro Ramadan, Pendell Meyers, Stephen W. Smith

**Affiliations:** 1 Instituto Dante Pazzanese de Cardiologia São Paulo SP Brasil Instituto Dante Pazzanese de Cardiologia, São Paulo, SP – Brasil; 2 Carolinas Medical Center Department of Emergency Medicine Charlotte NC EUA Carolinas Medical Center – Department of Emergency Medicine, Charlotte, NC – EUA; 3 Department of Emergency Medicine and University of Minnesota Hennepin Healthcare Minneapolis MN EUA Hennepin Healthcare, Department of Emergency Medicine and University of Minnesota, Minneapolis, MN – EUA

**Keywords:** Eletrocardiografia, Oclusão Coronária, Infarto do Miocárdio

## Abstract

Embora o modelo existente de classificação do infarto agudo do miocárdio (IAM) em IAMCSST e IAMSSST tenha sido benéfico, considera-se hoje que ele falha em abordar a complexidade das síndromes coronarianas agudas.

O estudo tem como objetivo examinar o atual paradigma IAMCSST-IAMSSST e defender um modelo mais detalhado, chamado de oclusão coronariana aguda (OCA) e Ausência de Oclusão Coronária Aguda (NOCA), para um diagnóstico e um manejo do IAM mais precisos.

Realizou-se uma análise abrangente da literatura médica existente, com foco nas limitações do modelo IAMCSST-IAMSSST. O estudo também descreve uma nova abordagem diagnóstica para pacientes apresentando do torácica nos departamentos de emergência.

O modelo IAMCSST-IAMSSST tradicional falha em prover um diagnóstico preciso e um tratamento efetivo, principalmente na identificação de oclusões da artéria coronária. O modelo OCA-NOCA é mais preciso em termos anatômicos e fisiológicos, e apoiado por pesquisa clínica extensa e opiniões de especialistas. Ele destaca a necessidade de rápida realização de eletrocardiogramas (ECGs) e terapias de reperfusão para casos suspeitos de OCA, visando melhorar os desfechos dos pacientes.

O modelo OCA-NOCA abre um novo caminho para pesquisas e aplicações clínicas futuras. Ele defende um entendimento mais abrangente dos mecanismos subjacentes das síndromes coronarianas agudas, levando a planos individualizados de tratamentos. Espera-se que essa nova abordagem incite novos debates e pesquisas acadêmicas, principalmente na área de cardiologia no Brasil, com o objetivo de aumentar a precisão diagnóstica e a eficácia do tratamento de pacientes com IAM.

## Introdução

O diagnóstico de infarto agudo do miocárdio (IAM) está em um momento decisivo. O modelo IAMCSST-IAMSSST (infarto agudo do miocárdio com supra de ST - infarto agudo do miocárdio sem supra de ST) tem sido fundamental no direcionamento de tratamentos urgentes de reperfusão e na orientação de profissionais médicos para tratar eventos coronários agudos. Embora o modelo tenha melhorado significativamente a sobrevida e a qualidade de vida do paciente, com o início da era da reperfusão no final do século passado, suas limitações em abordar as complexidades das doenças coronárias agudas têm sido cada vez mais evidentes.

Neste artigo, nós, cardiologistas do Instituto Dante Pazzanese de Cardiologia, em colaboração com os proponentes originais deste paradigma, fazemos essa afirmação, traçando paralelos entre os paradigmas atuais e mudanças seminais na literatura. Questionamos o paradigma IAMCSST-IAMSSST predominante no diagnóstico de IAM, e defendemos o uso dos termos infarto do miocárdio com oclusão, ou "oclusão coronariana aguda" (OCA) e ausência de oclusão coronária aguda (NOCA). Nós propomos este conceito como uma fundamentação anatômica e fisiológica mais precisa para o manejo e a classificação do IAM. Defendemos fortemente essa nova perspectiva com base em uma extensa pesquisa clínica, opiniões de especialistas, e em nossa experiência clínica. Nosso objetivo é estimular discussões cruciais na área de cardiologia no Brasil sobre a atualização de estratégias para o tratamento de IAM.

### Uma breve história do paradigma IAMCSST e IAMSSST

Infelizmente, e diferentemente de outras doenças, o IAM tem sido classificado somente de acordo com achados eletrocardiográficos individuais e não na sua fisiopatologia. No entanto, o paradigma IAMCSST-IAMSSST, que substituiu a terminologia "infarto do miocárdio com onda Q" e "infarto do miocárdio sem onda Q" cunhada em 2000, marcou um avanço significativo na era da terapia com reperfusão. Ele possibilitou a identificação precoce de pacientes em risco de morte do miocárdio antes do desenvolvimento de uma onda Q. Naquela época, a trombólise era o método de reperfusão disponível. Uma metanálise do início da era dessa terapia revelou um Número Necessário para Tratar (NNT) de 56 para o uso de fibrinolíticos. Quatro desses estudos, incluindo o estudo ISIS-2, não exigiram a presença de alterações eletrocardiográficas para a inclusão dos pacientes.^1 – 3^ Porém, análises de subgrupos encontraram uma associação com a Elevação do segmento ST (EST), mal definida, levando à melhora dos desfechos com trombolíticos (principalmente estreptoquinase).

**Figure:**
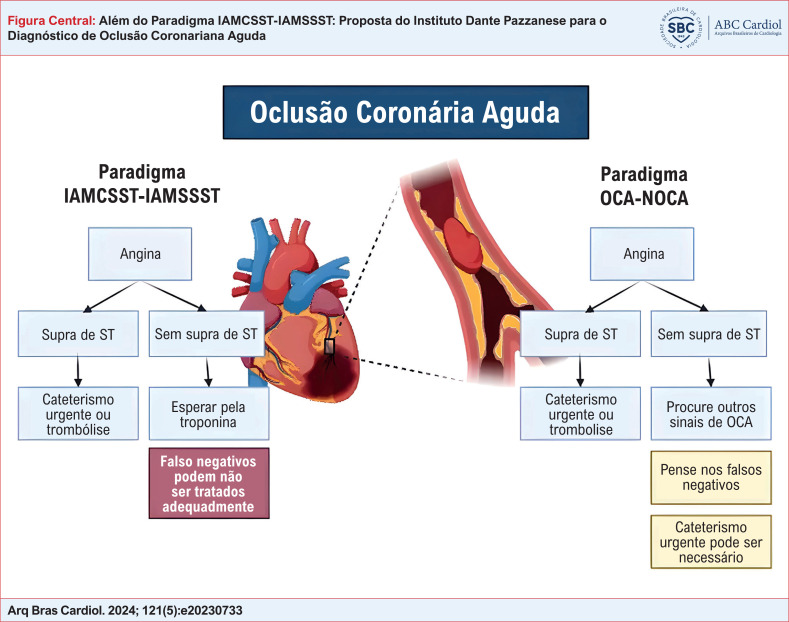


### A falsa dicotomia IAMCSST vs. IAMSSST: evidências crescentes

O paradigma IAMCSST foi um marco importante na medicina cardiovascular. Contudo, o esforço para padronizar os critérios IAMCSST expôs lacunas com consequências consideráveis para os pacientes.

Em uma tentativa de reconciliar vários critérios de EST, Menown et al.^4^ utilizaram a análise de regressão logística em um estudo caso-controle com 190 indivíduos, para determinar os pontos de corte ótimos: ≥ 2 mm em pelo menos uma derivação anterosseptal (V1–4) ou ≥ 1 mm nas demais derivações. Esse estudo usou o CK-MB para confirmar o Infarto do Miocárdio (IM) em vez de evidências angiográficas de OCA.^4^ Essa abordagem metodológica impossibilitou a distinção entre OCA e NOCA, resultando em uma sensibilidade de somente 56% para o diagnóstico de IAM com base nos biomarcadores.

Em 2004, Macfarlane et al.,^5^ em um estudo caso-controle, sugeriram pontos de corte de EST específicos para idade e sexo, aplicando técnicas estatísticas análogas às usadas em estudos prévios. Contudo, o desfecho primário para a confirmação diagnóstica foi novamente baseado nos valores de CK-MB, ignorando-se a confirmação diagnóstica de OCA.^5^ Com base nesses estudos caso-controle, a *American Heart Association* , a *American College of Cardiology Foundation* , e a *Heart Rhythm Society* (AHA/ACCF/HRS) redefinem, periodicamente, o que hoje se conhece por "critérios IAMST". Essa definição continua a reverberar nas diretrizes posteriores, incluindo a Quarta Definição Universal de IM.^6^

Pouco tempo depois que se chegou ao consenso de 2000, dúvidas surgiram acerca da adequação do paradigma IAMCSST-IAMSSST. O rótulo "IAMCSST", inadvertidamente, enfatizou somente um aspecto de um teste diagnóstico – a presença de EST no eletrocardiograma (ECG).^7 – 9^ Alguns médicos, possivelmente sem saber da origem do paradigma IAMCSST-IAMSSST, podem erroneamente acreditar que pacientes com OCA, mas sem EST no ECG, não se beneficiam da terapia de reperfusão.

Em 2001, Schmitt et al.^10^ estavam entres os primeiros que estudaram o paradigma IAMCSST-IAMSSST angiograficamente. Os autores encontraram que 29% dos 418 pacientes com OCA não preenchiam os critérios de IAMCSST. Particularmente, 50% dos ECGs dos pacientes com oclusão da artéria circunflexa não preencheram os critérios.^10^

Em uma análise^11^ post-hoc do ensaio PARAGON-B, 528 (27%) dos 1957 pacientes diagnosticados com Síndromes Coronarianas Agudas (SCAs) sem EST apresentavam oclusão total do vaso culpado. Esses pacientes apresentavam maior área infartada e maior mortalidade em seis meses ajustada quanto ao risco.

O estudo TRITON-TIMI-38^12^ apresentou outra evidência: dos 1198 pacientes com SCAs sem EST e Depressões do segmento ST (DSTs) isoladas, 314 (26,2%) apresentaram artérias culpadas completamente ocluídas. Koyama et al.^13^ também contribuíram para esse entendimento, encontrando que, nos casos de IAMSSST, 57% apresentavam fluxo TIMI 0, enquanto entre os pacientes com IAMSSST, 47% apresentavam fluxo TIMI-0, com uma taxa de mortalidade em torno de 5% em ambos os grupos.^13^ É evidente que IAMSSST com artérias coronárias ocluídas são essencialmente equivalentes ao IAMSSSTem termos de gravidade e desfechos.

Em 2021, Meyers et al.^14^ compararam o paradigma IAMCSST/IAMSSST com o modelo OCA/NOCA. Seu objetivo era identificar diferenças no tempo de cateterismo e desfechos relacionados entre OCA com IAMCSST e OCA sem IAMCSST. Os autores encontraram que 28% dos pacientes com IAMSSST apresentaram OCA detectada no cateterismo tardio, e 45% dos pacientes com OCA não preencheram os critérios para IAMCSST. O tamanho da área infartada desses pacientes OCA sem IAMCSST foi estatisticamente igual à dos pacientes com OCA e IAMCSST^14^ Em outro estudo examinaram a acurácia de marcadores eletrocardiográficos específicos de OCA em comparação à dos critérios para IAMCSST vigentes. Esses marcadores para OCA pré-definidos foram bem mais sensíveis e mantinham uma alta especificidade. Tais dados sugerem que uma interpretação precisa do ECG pode identificar rapidamente e de maneira não invasiva os pacientes com OCA e sem IAMCSST para reperfusão imediata.^15^

O estudo DIFOCCULT^16^ de 2020 apresentou evidências ainda mais convincentes. Cardiologistas, usando interpretação de experts do ECG e não somente critérios de IAMCSST, reclassificaram, de maneira cega, 28% dos pacientes com IAMSSST como OCA; esses pacientes apresentaram mortalidade em longo prazo significativamente maior que pacientes com IAMSSST classificados pelo ECG como apresentando NOCA. Uma metanálise da acurácia do teste diagnóstico, conduzido pela nossa equipe, oferece maiores esclarecimentos sobre os desafios diagnósticos associados com essa condição. Nós encontramos que a sensibilidade agrupada do supradesnivelamento do segmento ST detectar OCA foi somente 43,6% (9IC 95%: 34,7% - 52,9%), sugerindo que mais da metade dos casos de OCA pode não apresentar EST. A especificidade foi alta, de 96,5% (IC95%: 91,2%–98,7%). Outras análises empregando a estratégia OCA-NOCA demonstraram uma melhor sensibilidade, de 78,1% (IC 95%: 62,7%–88,3%) e uma especificidade similar de 94,4% (IC95%: 88,6%–97,3%).^17^

Em uma metanálise de prevalência, Khan et al.^18^ relataram que 25% dos 40 000 pacientes com IAMSSST encontravam-se com oclusão aguda da artéria no angiograma realizada no dia seguinte, sem circulação colateral. Em comparação aos pacientes com IAMSSST e uma artéria aberta, entre esses indivíduos, havia uma taxa de mortalidade quase duas vezes maior, mesmo sendo, em média, 15 anos mais jovens e apresentando menos comorbidades.^18^

Em outra metanálise de Hung et al.,^19^ 34% dos 60 000 pacientes com IAMSSST apresentaram oclusão total da artéria coronária. Em comparação aos pacientes com uma artéria aberta, os pacientes com OCA apresentaram, ajustados quantos aos outros fatores clínicos: fração de ejeção mais baixa, valores mais altos de biomarcadores, mais choque cardiogênico, e mortalidade mais alta (OR 1,72, IC95% 1,49-1,98; p < 0,001).^19^

O paradigma IAMCSST-IAMSSST, ao definir uma doença somente por um aspecto impreciso (EST) de um teste (ECG), negligencia a real fisiopatologia da OCA. Isso resulta no paradoxo do "sem falso negativo": se um paciente tem OCA, sem EST, não há IAMCSST e, portanto, não há teste falso negativo para IAMCSST. Isso resultou na exclusão de pacientes com OCA sem IAMCSST dos bancos de dados e, assim, em dados padronizados limitados sobre a sensibilidade e a especificidade de a EST em diagnosticar OCA. Essa lacuna na literatura dificulta a tomada de decisão clínica, uma vez que os médicos precisam usar critérios potencialmente inadequados para abordar a Síndrome Coronariana Aguda (SCA).

### Urgência no tratamento das artérias coronárias ocluídas: "tempo é miocárdio"

Na SCA, a frase "tempo é miocárdio" aplica-se aos pacientes com OCA. Para esses, esperar não é uma opção; a terapia de reperfusão imediata é imperativa. Os níveis de troponina, embora comumente usados no diagnóstico de IM, não são eficazes no diagnóstico de OCA em situações agudas pelas seguintes razões;

- Demora: os resultados de troponina não são instantâneos; eles demoram tanto para serem adquiridos (coleta de sangue) quanto para serem processados; enquanto isso, o miocárdio está em risco;
- Falta de sensibilidade: a troponina ultrassensível (us) inicial não é sensível para OCA; em um estudo, a troponina I-us foi menos sensível que o limiar de referência para IM tipo I (52 ng/L) em 27% dos IAMCSST.^20^ Em uma segunda população da vida real, o valor da troponina inicial esteve abaixo que o limite superior de referência em 47% dos pacientes com IAMCSST.^21^ Ainda, os níveis de troponina não distinguem lesão miocárdica de IAM, IM do tipo 1 do IM do tipo 2, nem OCA de NOCA. Consequentemente, a troponina-us não é um marcador confiável para a tomada de decisão clínica imediata.

Talvez a objeção mais comum ao fato de que pacientes com OCA e IAMSSST requeiram reperfusão de emergência deriva de ensaios randomizados comparando intervenções imediatas *vs* . tardias em pacientes com IAMSSST e não mostrando nenhum benefício. No entanto, esses estudos são amplamente mal interpretados. Entre esses estudos, o mais notável é o estudo TIMACS.^22^ Nesse estudo, o grupo submetido à "intervenção precoce" teve um tempo mediano de intervenção de 16 horas, muito longo para oferecer qualquer benefício a pacientes com OCA. Ainda, o TIMACS não inclui pacientes sintomáticos. Contudo, todos os estudos que definiram intervenção precoce como menos de duas horas e incluíram pacientes sintomáticos demonstraram benefício da intervenção precoce.^22 – 29^

Além disso, o estudo TIMACS e outros estudos são geralmente mal caracterizados por envolver somente pacientes com IAMSSST. No entanto, esse e outros estudos similares também incluíram pacientes sem níveis elevados de troponina, quem, por definição, apresentavam Angina Instável (AI), e não IAM. Sabe-se que níveis elevados de troponina são um forte preditor de um risco elevado de SCA. No caso do TIMACS, 22% dos 3031 pacientes foram diagnosticados com AI, e não IAM. Vale notar que os pacientes com troponina negativa, indicativo de AI, nunca pareceram se beneficiar de um tratamento urgente em vez de um tratamento tardio. Como uma reperfusão mais rápida poderia levar a um tamanho reduzido da área infartada, se não há nenhuma área infartada? Consequentemente, a falta de uma diferença perceptível no desfecho primário não é surpreendente, uma vez que 22% da população do estudo não apresentaram mecanismo de benefício plausível.

Apesar de um tempo médio de espera de 16 horas para cateterismo no grupo da intervenção "precoce" no TIMACS, observou-se uma tendência notável para um benefício no desfecho primário, com uma redução de 2,1% no risco absoluto de morte, IM, e acidente vascular cerebral (9,4% vs. 11,5%, RR 0,81, IC95% 0,63-1,04). Isso foi observado apesar do tempo maior de espera para o grupo intervenção "precoce" e a inclusão de 22% de pacientes com AI. Ainda, o subgrupo com escore GRACE > 140 mostrou benefícios em todos os desfechos.

Um importante ensaio subsequente, frequentemente citado por aqueles que afirmam não haver vantagem em relação ao manejo mais precoce do IAMSSST, é o ensaio VERDICT.^29^ Entre os 2147 pacientes incluídos, 80% foram diagnosticados com IAMSSST, e 20% apresentaram AI sem IAM. Novamente, a inclusão de AI dilui os resultados. Seguindo todas as diretrizes, o estudo excluiu pacientes apresentando dor. No VERDICT, os tempos de angiograma para os grupos precoce e tardio foram 4,7 e 62 horas, respectivamente. Embora 4,7 horas seja uma melhora significativa em comparação às 16 horas no grupo precoce do TIMACS, o tempo continua um atraso considerável no tratamento do IM /OCA, um fato prontamente reconhecido no contexto do manejo do IAMSSST. Não foi surpresa que o desfecho primário, que considerou os potenciais benefícios de um rápido angiograma para uma coorte composta tanto por pacientes com IAMSSST assintomáticos como por pacientes com AI assintomáticos e troponinas seriadas negativas, não produziu uma vantagem perceptível no grupo precoce.

No entanto, assim como o ensaio TIMACS,^22^ no VERDICT,^29^ o subgrupo com um escore GRACE superior a 140 mostraram benefícios significativos no desfecho primário. Outras análises do grupo de pacientes com IAMSSST revelaram uma redução absoluta de 4% no desfecho primário composto (morte, IAM, e reinternações por isquemia ou insuficiência cardíaca). Essa diferença não foi estatisticamente significativa, pois o estudo não discerniu uma variação de 4% (28,8% vs. 32,7%, RR 0,85, IC95% 0,71-1,01). Uma intervenção demonstrando uma redução no risco absoluto de 4% (NNT=25) em um desfecho tão centralizado no paciente teria uma importância clínica se validada por um estudo com poder suficiente. Assim, os resultados do estudo VERDICT não contradizem essa proposição.

Portanto, os estudos mais extensos e pertinentes^22 , 29^ indicam, consistentemente, benefícios significativos em subgrupos de pacientes com IAMSSST de alto risco. Os estudos também sugerem que os benefícios observados no grupo inteiro de pacientes com IAMSSST poderiam ser confirmados se eles tivessem poder suficiente. Esses resultados originam-se de populações em que os pacientes relatavam estar assintomáticos. Além disso, os tempos de intervenção precoce, 16 horas^22^ e 4,7 horas^29^ foram substancialmente mais longos do tempo tipicamente considerado em uma abordagem de emergência (definido como inferior a 90 minutos). De fato, outro ensaio, o RIDDLE-IAMSST, em que o tempo para Intervenção Coronária Percutânea (ICP) foi genuinamente mais cedo (1,4 horas), demonstrou que uma estratégia invasiva imediata em pacientes com IAMSSST associa-se a taxas mais baixas de morte ou novo IM em comparação a uma estratégia invasiva tardia.^30^ Tal discrepância sugere o potencial para mais benefícios se a intervenção tivesse sido genuinamente de emergência, e principalmente se o estudo tivesse sido limitado a pacientes com OCA e IAMSSST com achados específicos no ECG e geralmente sintomáticos (por exemplo, dor torácica persistente). Assim, nossa posição não é defender o manejo emergente de todos os casos de IAMSSST principalmente aqueles com sintomas resolvidos. Em vez disso, nós enfatizamos a importância de reconhecer e tratar prontamente subgrupos de pacientes com IAMSSST e em mais alto risco: os pacientes com OCA. Esses indivíduos, que sofreram uma oclusão ou semi oclusão aguda dos vasos culpados, podem se beneficiar do manejo de emergência. A maioria pode ser identificada por ECG usando características além da EST.

Diante da crítica realidade anatômica de uma artéria ocluída, recomenda-se que o tempo da terapia de reperfusão para pacientes com OCA seja alinhado com os tempos porta-agulha e porta-balão padronizados para os pacientes com IAMCSST. Qualquer demora em se buscar clareza diagnóstica pode levar a danos irreversíveis no miocárdio e um impacto negativo sobre os desfechos.

### O novo paradigma: OCA-NOCA

Dadas as limitações inerentes ao paradigma IAMCSST-IAMSSST, uma abordagem mais cuidadosa foi proposta, focando nos detalhes anatômicos e fisiológicos da SCA. Esse paradigma distingue os pacientes em duas categorias:

- OCA: Essa designação aplica-se a pacientes com OCA ou quase oclusão, com circulação colateral limitada, colocando-os em risco imediato de IAM transmural. Essa condição causa predominantemente IM tipo 1. A necessidade de terapia de reperfusão para esses pacientes é urgente, independentemente dos achados do ECG. A principal preocupação é a obstrução anatômica e seu risco associado, e não os detalhes dos critérios eletrocardiográficos. Apesar da importância do ECG, outros achados clínicos e diagnósticos, tais como anormalidades na motilidade da parede no ecocardiograma ou obstrução de vaso detectada por angiografia por tomografia computadorizada, pode auxiliar no diagnóstico da OCA, principalmente se o ECG for inconclusivo.
- NOCA: Essa categoria inclui pacientes sem oclusão coronária. Porém, uma vez que a NOCA pode envolver placa rompida instável, os pacientes mantêm-se vulneráveis à potencial trombose e isquemia coronária. Eles podem apresentar níveis elevados de troponina ou alterações no ECG, como inversão da onda T. Embora esses pacientes necessitem de uma angiografia ou de uma intervenção caso um vaso culpado seja identificado, o ECG não é sensível para NOCA mas, felizmente, ele não precisa ser, pois não são necessárias intervenções imediatas, e o diagnóstico pode aguardar pela medida da troponina.

Essa classificação busca melhorar nosso entendimento e o manejo da SCA. Ela permite que os médicos enfatizem não só o ECG, mas também a fisiopatologia subjacente, alinhando, de maneira mais próxima, os tratamentos às necessidades individuais do paciente ( Figura Central ). Meyers e Smith defenderam esse paradigma no seu "Manifesto OMI", que apresenta um direcionamento importante às pesquisas iminentes e à aplicação clínica.

Avanços na tecnologia têm influenciado significativamente o refinamento do manejo do IAM. Algoritmos de inteligência artificial mais recentes exibem alta precisão diagnóstica e estão em rápida evolução. Tais avanços poderiam melhorar o paradigma OCA-NOCA, fornecendo aos médicos ferramentas diagnósticas ainda mais precisas.^31 , 32^

### Outros sinais eletrocardiográficos da OCA

**Figura 1:**
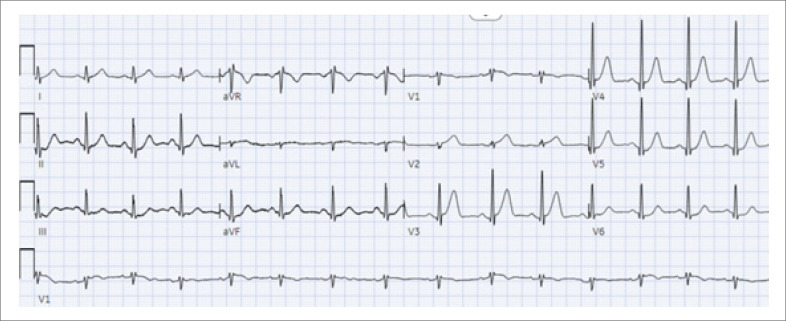
Ondas T hiperagudas; eletrocardiograma a 25 mm/s mostrando uma onda T hiperaguda nas derivações V2-V4.

No cenário em evolução do diagnóstico da OCA, um dos sinais eletrocardiográficos chave é a onda T hiperaguda, caracterizada por uma área sob a onda T aumentada em relação ao complexo QRS, incluindo uma base larga, comprimento e convexidade aumentadas, e uma tendência à simetria. Visualmente, a onda T parece se expandir em todas as direções – aproximando-se e se afastando da onda T, e para cima – resultando em um alargamento do complexo QT e um pico mais arredondado ( Figura 1 ).

Ondas T invertidas não são indicativas de fases hiperagudas de infarto, como alguns podem presumir a partir do termo "isquemia subepicárdica". Ondas T invertidas registradas por derivações sobre o miocárdio isquêmico aparecem após a reperfusão aguda, e em estados isquêmicos crônicos ou subagudos, ou reciprocamente às ondas T hiperagudas em uma derivação oposta. Quando ondas T negativas estão presentes, o problema pode não estar localizado na zona subepicárdica das derivações sobrejacentes, e sim em zonas mais distantes, no miocárdio oposto.

**Figura 2:**
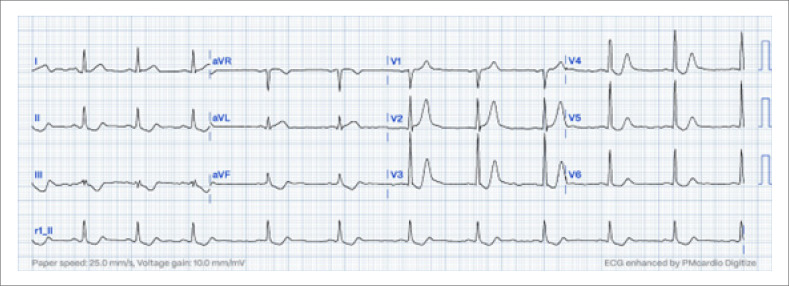
Sinal sutil de De Winter; eletrocardiograma a 25 mm/s mostrando o padrão de De Winter, caracterizado pela depressão do segmento ST no ponto J nas derivações V1-V2 a V6, seguida por um ST ascendente e uma curva T positiva, simétrica, hiperaguda.

Além desses sinais, destaca-se o padrão "De Winter". Estima-se que esse padrão ocorra em somente 2% dos pacientes com IM anteroapical submetidos à angioplastia primária. No contexto clínico de dor torácica isquêmica aguda, o padrão eletrocardiográfico De Winter exibe uma DST medida no ponto J de pelo menos 1 mm nas derivações V1-V2 à V6, seguido por um segmento ST geralmente na ascendente, e uma onda T hiperaguda alta, positiva e simétrica ( Figura 2 ). Apesar de as ondas T de Winter receberem muita atenção, elas não são mais importantes que a ondas T hiperagudas, das quais correspondem a somente uma pequena fração.

**Figura 3:**
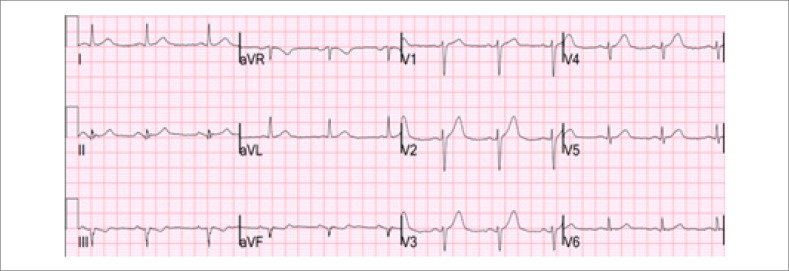
Elevação do segmento ST na parede ântero-apical; eletrocardiograma mostrando uma sutil elevação do segmento ST de aproximadamente 1 mm nas derivações V1-V4, o que não preenche o critério estabelecido pela Definição Universal de Infarto do Miocárdio; o uso da calculadora de quatro variáveis indicou oclusão coronariana aguda (OCA); a angiografia coronariana subsequente confirmou oclusão da artéria descendente anterior esquerda.

Fazendo a transição das ondas T, a distorção da porção terminal do complexo QRS oferece outra pista diagnóstica. Definida como a ausência de uma onda S abaixo da linha isoelétrica TP e a ausência de uma onda J na derivação V2 ou V3, ela é um achado muito específico da Artéria Descendente Anterior Esquerda (ADAE) em comparação à repolarização precoce.^33^ Para diferenciar EST isquêmico do fisiológico na V2 e V3, a fórmula de Smith de quatro variáveis pode ser usada, com 88,8% de sensibilidade e 94,7% de especificidade ( Figura 3 ).^34 , 35^ A calculadora está disponível em: Subtle Anterior STEMI Calculator (4-Variable) (mdcalc.com). Outro sinal eletrocardiográfico de OCA é o padrão Aslanger, que está associado com infarto da parede inferior e Doença Arterial Coronariana (DAC) de múltiplos vasos, não preenchendo os critérios clássicos para EST em duas derivações contíguas. Ele é caracterizado pela EST somente na derivação III. E DST em qualquer das derivações V4-V6, mas não em V2, e um segmento ST em V1 maior que em V2.^36^

Outro sinal eletrocardiográfico de OCA envolve a oclusão do ramo diagonal, afetando a parede anteromedial do coração. De acordo com a atual terminologia das paredes do IM, a qual se baseia na ressonância magnética, as derivações I, aVL e V2 (e ocasionalmente V3) correspondem à parede anteromedial do ventrículo esquerdo. Em alguns casos, a EST pode ser evidente só em aVL e V2.^37 , 38^ Essa região é suprida pelo primeiro ramo diagonal da ADAE. Uma falta de conhecimento dessa terminologia poderia levar cardiologistas a pensarem que aVL e V2 não são derivações contíguas. Quando essas derivações são afetadas, uma mudança recíproca geralmente ocorre na parede inferior, particularmente na derivação III. Esse padrão específico foi denominado padrão "bandeira da África do Sul".

O inverso também parece ser verdadeiro: qualquer depressão do segmento ST em aVL auxilia no diagnóstico OCA na parede inferior versus pericardite. Um estudo envolvendo 426 pacientes com IM inferior e complexo QRS normal encontrou que 99% das elevações inferiores do segmento ST exibiram algum grau de DST recíproco na derivação aVL. Isso ocorreu mesmo quando a EST foi sutil (menos que 1mm) e quando havia EST em V5 e em V6. Por outro lado, na pericardite, não se observou DST em nenhuma das derivações exceto aVR.^39^ Outra importante alteração a ser considerada é DST nas derivações V1 a V3/V4, que serve como um indicador recíproco de SE nas derivações V7 a V9, correspondendo à parede lateral do coração.^31 , 40 , 41^

O diagnóstico de IAM torna-se particularmente desafiador quando o paciente apresenta bloqueio de ramo esquerdo (BRE).^42^ As diretrizes do ACC/AHA de 2013^43^ e as diretrizes do ESC STEMI de 2017 e 2023^44^ recomendam que pacientes com suspeita clínica de isquemia miocárdica e BRE sejam tratados independentemente de o BRE ser previamente conhecido ou não. Um fato importante é que as diretrizes enfatizam a presença de um BRE novo ou presumidamente novo em si não prediz IM.^43 – 45^ Sgarbossa et al.^46^ propuseram um sistema de escore que foi posteriormente refinado pela introdução de um critério proporcional, conhecido como critério de Smith modificado, também validado para ritmo ventricular estimulado.^47 , 48^

Segundo a Quarta Definição Universal de IM,^6^ um bloqueio de ramo direito (BRD) novo ou presumidamente novo sem alterações no segmento ST ou onda T associadas deve ser considerado como um ECG equivalente à IAMCSST. Essas recomendações são primariamente baseadas em um estudo retrospectivo de Widimsky et al.,^49^ que incluiu 6742 pacientes com IM agudo.^49^ Nesse estudo, nem todos os pacientes foram submetidos à angiografia de emergência como um protocolo de ICP primária. Outras evidências desafiam o BRE como um indicador de angiografia coronária de emergência, uma vez que a probabilidade de IM foi similar à de pacientes sem bloqueio.^50^ Assim, mais dados de desfechos são necessários para pacientes apresentando dor torácica, BRD presumidamente novo, e sem desvio importante de ST.

**Figura 4:**
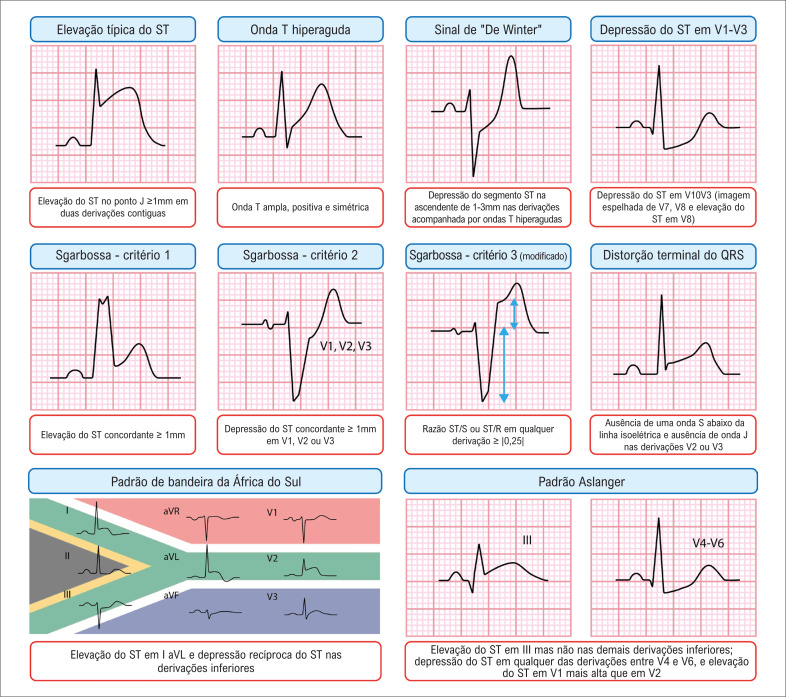
Indicadores eletrocardiográficos chave para oclusão coronariana aguda.

Para apresentar uma visão sucinta de vários sinais e achados discutidos, a Figura 4 resume esses elementos para referência e melhor compreensão.

### Diretrizes atuais e sua abordagem binária ao diagnóstico de IM agudo

As diretrizes atuais para o manejo de IAM são meticulosamente elaboradas, servindo como base para o tratamento de milhares de pacientes por cardiologistas e clínicos gerais. Essas diretrizes afirmam que, se um paciente com OCA, mas sem critérios eletrocardiográficos para IAMCSST, chega no departamento de emergência, é improvável que esse paciente seja submetido a cateterismo de urgência dentro do tempo porta-balão recomendado para IAMCSST.^51^ Em vez disso, os médicos tipicamente solicitam um teste de troponina, que pode levar de uma a duas horas para ficar pronto. Durante esse intervalo, o paciente geralmente recebe medicamentos antianginosos, inclusive morfina. Embora novos testes de troponina-us forneçam rápidos resultados,^52^ um nível elevado de troponina confirma um IAM. Se a história clínica seja sugestiva de IM tipo 1, a troponina ainda não diferencia OCA de NOCA. Quando o nível de troponina aumenta o suficiente para indicar OCA, uma lesão miocárdica substancial irreversível já ocorreu, e a janela crítica para o tempo porta-agulha e tempo porta-balão já está geralmente fechada. Assim, o diagnóstico de OCA deve ser determinado antes de os resultados de troponina estiverem disponíveis por meio de interpretação do ECG por experts ou uso de inteligência artificial na eletrocardiografia. A morfina pode confundir o diagnóstico ao levar a um falso senso de segurança, uma vez que o paciente está aparentemente sem dor (mas não sem isquemia). Portanto, o uso de morfina pode estar associado a um tempo mais longo para realização da angiografia.^53^

As diretrizes brasileiras para IAMSSST recomendam estratégias invasivas urgentes para pacientes que apresentam dor torácica recorrente ou refratária.^33^ Essa abordagem serve como uma rede de segurança para pacientes com OCA que não preenchem critérios eletrocardiográficos para IAMCSST. Recomenda-se cateterismo se um paciente apresenta um ECG falso negativo, mas continua com dor torácica. Embora essa recomendação seja muito prudente, vale notar que sua implementação não é tão disseminada como pode-se esperar, mesmo em locais em ela é oficialmente endossada.^51^

**Figura 5:**
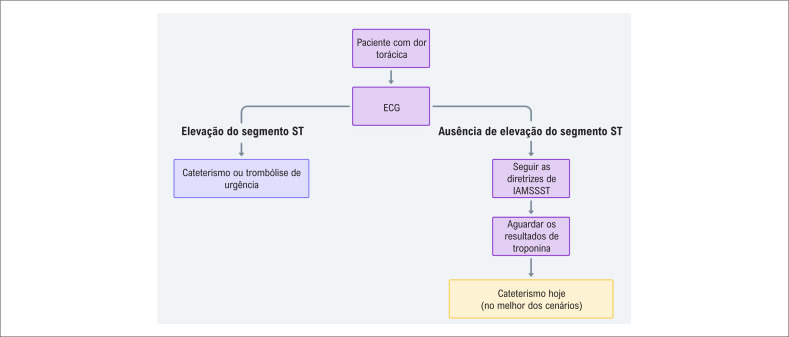
Fluxograma ilustrando a abordagem atual para o diagnóstico de infarto agudo do miocárdio; essa tem sido o pilar para o tratamento de muitos pacientes em várias áreas médicas; o algoritmo retrata as limitações de se confiar somente no eletrocardiograma e nos níveis de troponina.

Na OCA, a troponina inicial pode ser positiva ou negativa, similar a alguns casos óbvios de IAMCSST. Geralmente, o cateterismo para IAMSSST é adiado para uma data futura; com sorte, ele pode ocorrer em um momento mais tarde no mesmo dia. No entanto, ambos os cenários não se alinham aos prazos recomendados pelas diretrizes atuais ( Figura 5 ).

Nós somos gratos pela atenção e pelo rigor aplicados no desenvolvimento dessas diretrizes. No entanto, acreditamos que haja uma oportunidade para uma ação mais assertiva nessa área. Nós defendemos a introdução de um novo paradigma para a abordagem do IM. Esse paradigma visa ser mais inclusivo e preciso, fornecendo um melhor suporte para cardiologistas, clínicos gerais, e estudantes de medicina no futuro.

### Nossa proposta de abordagem para pacientes com dor torácica no departamento de emergência: O paradigma OCA-NOCA

Considerando a necessidade urgente de um diagnóstico rápido e preciso de IAM, nós propusemos uma abordagem refinada que enfatiza a diferenciação entre OCA e NOCA. Se um paciente com fatores de risco epidemiológicos apresenta-se no departamento de emergência com angina (ou outros sintomas isquêmicos), o paciente deve ser imediatamente avaliado quanto à potencial OCA ( Figura Central ). Essa abordagem é delineada para ser tanto prática como efetiva, reconhecendo as complexidades inerentes e as limitações no diagnóstico de IAM.

Processo diagnóstico: passo-a-passo

**Figura 6:**
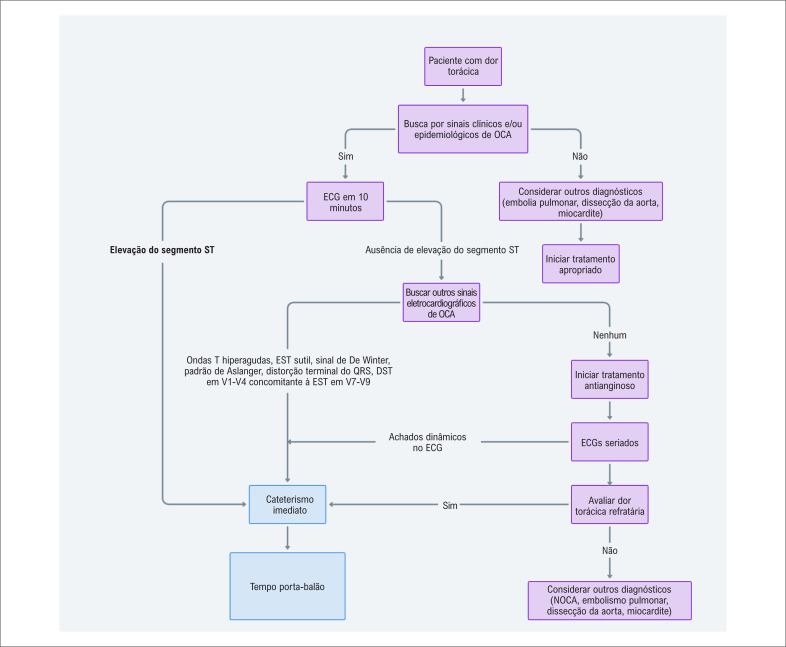
Algoritmo de decisão proposto para o manejo de dor torácica no departamento de emergência; o fluxograma ilustra uma abordagem abrangente para a avaliação e o manejo de pacientes apresentando dor torácica no departamento de emergência. O algoritmo incorpora critérios diagnósticos tradicionais bem como critérios detalhados, visando melhorar a identificação e o tratamento de Oclusão Coronariana Aguda (OCA); etapas chave incluem avaliação clínica inicial, rápida avaliação por Eletrocardiograma (ECG), e consideração tanto de Elevação do Segmento ST (EST) como outros sinais eletrocardiográficos. O algoritmo também considera a possibilidade de casos falso-negativos de OCA, enfatizando a importância de monitorar dor torácica ou alterações dinâmicas no ECG como indicadores de terapia de reperfusão imediata. Importante notar que, enquanto esse algoritmo proposto oferece um potencial avanço no manejo de dor torácica, sua implementação em uma escala nacional, particularmente no Sistema Único de Saúde no Brasil, pode aumentar o número de intervenções coronárias percutâneas. Portanto, as implicações logísticas e econômicas dessa proposta devem ser cuidadosamente consideradas, e posteriormente validadas por ensaios clínicos randomizados antes de sua ampla adoção; DST: depressão do segmento ST.

1. Avalição eletrocardiográfica imediata: na chegada no departamento de emergência, pacientes com fatores de risco epidemiológicos e sintomas sugestivos de isquemia (angina ou equivalente) devem ser submetidos a um ECG nos primeiros dez minutos. Essa rápida avaliação é crucial na identificação de potenciais casos de OCA.
2. Pesquisa por EST no ECG: uma etapa chave na avaliação de um paciente com suspeita de IAM no departamento de emergência é a avaliação da EST no ECG. Enquanto a presença de uma elevação significativa do ST é um forte indicador de OCA, é crucial compreender que aproximadamente 30-50% dos casos de OCA podem não apresentar esse sinal clássico. Isso destaca a importância de não se confiar somente na EST no diagnóstico de OCA. Os médicos devem estar cientes dessa possibilidade e preparados para maior investigação, mesmo na ausência de elevação importante do segmento ST, para assegurar que os casos de OCA não sejam negligenciados.
3. Procura por outros sinais de OCA: além de se avaliar a EST, médicos devem estar atentos para identificar outros indicadores eletrocardiográficos que possam sugerir OCA:^54 , 55^ EST sutil (< 1mm) é frequentemente vista na OCA, e qualquer EST ≥ 1mm na V2-V4 pode ser normal ou por OCA na ADAE; usar a fórmula de quatro variáveis para diferenciar;^34^ ondas T hiperagudas com EST sutil com ou sem EST,^56^ sinal de De Winter,^57 , 58^ padrão de Aslanger,^36 , 59 , 60^ DST em V1-V4 representando alterações recíprocas da parede lateral (V7-V9),^41^ distorção terminal do QRS,^33^ EST nas derivações inferiores acompanhada por qualquer DST na aVL indicativo de alterações recíprocas a partir da parede anterior média,^39^ critério de Sgarbossa modificado por Smith em casos de BRE^47 , 61^ ou ritmo estimulado.^48^ A presença de qualquer desses sinais necessita reperfusão imediata. É fundamental reconhecer que uma OCA pode ocorrer mesmo sem esses sinais eletrocardiográficos específicos, ou mesmo com um ECG completamente normal.
4. ECGs seriados e comparação: na ausência de sinais claros de OCA, ECG seriados devem ser realizados, e ECGs prévios obtidos para comparação. Essa etapa é vital para identificar mudanças dinâmicas que possam indicar isquemia miocárdica em progresso.
5. Os pacientes devem receber tratamento com antianginosos. Deve-se evitar o uso de opioides para o alívio da dor até o paciente ser encaminhado para o laboratório de cateterismo, uma vez que a medicação esconderá sintomas isquêmicos. Um paciente com dor torácica persistente na SCA requer uma abordagem invasiva urgente, mesmo na ausência de sinais eletrocardiográfica na OCA. Paralelamente, é essencial avaliar outras potenciais causas de dor torácica que possam não responder à terapia antianginosa ( Figura 6 ).

Além dos sintomas isquêmicos, ECGs repetidos, e níveis de troponina, várias ferramentas diagnósticas podem servir como indicadores suplementares para identificar OCA. Tipicamente, a ecocardiografia revela anormalidade na motilidade da parede durante uma OCA; mas, são necessários contraste à base de microbolhas, técnica excelente, e interpretação por profissional experiente para uma alta sensibilidade. Finalmente, uma angiotomografia computadorizada coronariana de emergência deve ser facilmente acessível e análoga à abordagem atual de se avaliar pacientes com acidente vascular cerebral agudo no contexto de "oclusão de grandes vasos".^61^

Enquanto essa abordagem visa fornecer uma via clara e viável para o diagnóstico de IAM, reconhecemos que nenhum método diagnóstico é infalível. A complexidade das apresentações do IAM significa que sempre haverá casos em que o diagnóstico não é imediatamente claro. Nossa abordagem é delineada para maximizar a acurácia diagnóstica e, ao mesmo tempo, ser adaptável a nuances de apresentações individuais dos pacientes.

## Conclusão

O cenário do diagnóstico de IAM está prestes a sofrer uma mudança de paradigma. Enquanto o modelo IAMCSST-IAMSSST nos serviu bem como uma transição da era de reperfusão, evidências crescentes e observações clínicas indicam suas limitações em abordar detalhes mais complexos da SCA. Este artigo destaca essas restrições e apresenta o paradigma OCA-NOCA como uma estratégia mais precisa do ponto de vista anatômico e fisiológico no manejo do IAM. Antecipamos que esta abordagem refinada irá melhorar os desfechos do paciente otimizando a acurácia diagnóstica e maximizando a eficácia das terapias de reperfusão.

Apresentamos evidências desafiando a acurácia diagnóstica e terapêutica do paradigma IAMCSST-IAMSSST, destacando a taxa notável de casos negligenciados de infartos do miocárdio com oclusão e das limitações em se confiar somente no ECG (e especialmente na EST) e nos níveis de troponina para tomadas de decisões clínicas. Além disso, delineamos a abordagem do Instituto Dante Pazzanese de Cardiologia em diagnosticar pacientes com dor torácica no ambiente de emergência, reforçando a importância da rápida avaliação do ECG e da terapia de reperfusão imediata para casos potenciais de OCA.

Nós acreditamos que o paradigma OCA-NOCA oferece uma direção promissora para pesquisas e prática clínica futuras. Ela estimula médicos a complementarem testes diagnósticos convencionais com um maior entendimento da fisiopatologia subjacente da SCA, levando a tratamentos mais ajustados às necessidades individuais dos pacientes. Acreditamos que este artigo estimulará a discussão e a investigação na comunidade de cardiologistas brasileiros, o que aumentará a precisão diagnóstica e a eficácia do tratamento dos pacientes com IAM.
