# Supplementary material for: Além do Paradigma IAMCSST-IAMSSST: Proposta do Instituto Dante Pazzanese para o Diagnóstico de Oclusão Coronariana Aguda
Source: Arq Bras Cardiol. 2024 Jun 21;121(5):e20230733. [Article in Portuguese] doi: 10.36660/abc.20230733 (PMC11216332; doi:10.36660/abc.20230733)
Supplement: Translation

# Translation: Beyond STEMI-NSTEMI Paradigm: Dante Pazzanese's Proposal for Occlusion Myocardial Infarction Diagnosis

**DOI:** 10.36660/abc.20230733i

**Authors:** José Nunes De Alencar, Fausto Feres, Mariana Fuziy Nogueira De Marchi, Kleber Gomes Franchini, Matheus Kiszka Scheffer, Sandro Pinelli Felicioni, Ana Carolina Muniz Costa, Rinaldo Carvalho Fernandes, Hugo Ribeiro Ramadan, Pendell Meyers, Stephen W. Smith

**Affiliations:** 1 São Paulo SP Brazil Instituto Dante Pazzanese de Cardiologia, São Paulo, SP – Brazil; 2 Charlotte NC USA Carolinas Medical Center – Department of Emergency Medicine, Charlotte, NC – USA; 3 Minneapolis MN USA Hennepin Healthcare, Department of Emergency Medicine and University of Minnesota, Minneapolis, MN – USA

**Keywords:** Electrocardiography, Coronary Occlusion, Myocardial Infarction

## Abstract

Although the existing framework for classifying acute myocardial infarction (AMI) into STEMI and NSTEMI has been beneficial, it is now considered to be falling short in addressing the complexity of acute coronary syndromes.

The study aims to scrutinize the current STEMI-NSTEMI paradigm and advocate for a more nuanced framework, termed as occlusion myocardial infarction (OMI) and non-occlusion myocardial infarction (NOMI), for a more accurate diagnosis and management of AMI.

A comprehensive analysis of existing medical literature was conducted, with a focus on the limitations of the STEMI-NSTEMI model. The study also outlines a new diagnostic approach for patients presenting with chest pain in emergency settings.

The traditional STEMI-NSTEMI model falls short in diagnostic precision and effective treatment, especially in identifying acute coronary artery occlusions. The OMI-NOMI framework offers a more anatomically and physiologically accurate model, backed by a wealth of clinical research and expert opinion. It underscores the need for quick ECG assessments and immediate reperfusion therapies for suspected OMI cases, aiming to improve patient outcomes.

The OMI-NOMI framework offers a new avenue for future research and clinical application. It advocates for a more comprehensive understanding of the underlying mechanisms of acute coronary syndromes, leading to individualized treatment plans. This novel approach is expected to ignite further scholarly debate and research, particularly in the Brazilian cardiology sector, with the goal of enhancing diagnostic accuracy and treatment effectiveness in AMI patients.

## Introduction

The diagnosis of acute myocardial infarction (AMI) is at a pivotal juncture. For many years, the (STEMI), (NSTEMI), STEMI-NSTEMI (ST-elevated myocardial infarction-non-ST elevated myocardial infarction) model has been fundamental in guiding urgent reperfusion treatments and directing medical professionals to treat acute coronary events. Although the model has significantly improved patient survival and quality of life, since the beginning of the Reperfusion Era at the end of the last century, its limitations in addressing the complexities of acute coronary conditions have been increasingly evident.

In this article, Cardiologists from Instituto Dante Pazzanese de Cardiologia, in collaboration with the proponents of this new paradigm, present this statement, drawing parallels between current paradigms and seminal shifts in the literature. We question the prevailing STEMI-NSTEMI paradigm for AMI diagnosis, and we advocate for the terms occlusion myocardial infarction (OMI) and non-occlusion myocardial infarction (NOMI). Notably, in the Portuguese version of this paper, "oclusão coronariana aguda (OCA)" is the terminology used for OMI. We propose this concept as a more precise anatomical and physiological framework for managing and classifying AMI. We strongly advocate for this new perspective based on extensive clinical research, expert opinions, and our clinical experience. We aim to spark crucial discussions in the cardiology field in Brazil about updating strategies for AMI treatment.

### A brief history of the "STEMI" and "NSTEMI" paradigm

Unfortunately, and unlike other diseases, AMI has thus far always been classified only according to individual electrocardiogram (ECG) findings rather than the underlying pathophysiology. Nevertheless, the STEMI-NSTEMI paradigm, which replaced the previous "Q-wave myocardial infarction" and "non-Q-wave myocardial infarction" terminology in 2000, marked a significant advancement in the era of reperfusion therapy. It enabled the early identification of patients at risk of myocardial death before the development of a Q wave. At that time, thrombolysis was the primary reperfusion method available. A meta-analysis from the early era of this therapy revealed a number needed to treat (NNT) of 56 for the use of fibrinolytics. Notably, four of these studies, including the ISIS-2 study, did not require ECG alterations for patient inclusion.^1 – 3^However, subgroup analyses found an association with ST-segment elevation (STE), poorly defined, and improved outcomes with thrombolytics (primarily streptokinase).

### The false dichotomy of STEMI vs. NSTEMI: A growing body of evidence

The STEMI paradigm was a significant milestone in cardiovascular medicine. However, the effort to standardize the STEMI criteria has exposed gaps with considerable consequences for patients.

In an early attempt to reconcile various STE criteria, Menown et al.^4^used logistic regression analysis in a case-control study of 1190 subjects to determine the optimal STE cut-offs: ≥ 2 mm in at least one of the anteroseptal leads (V1–4) or ≥ 1 mm in any other leads. This study relied on CK-MB to confirm myocardial infarction (MI), instead of angiographic evidence of acute coronary occlusion (ACO).^4^This methodological approach meant the study could not distinguish ACO from non-ACO, resulting in a sensitivity of only 56% for diagnosing AMI based on biomarkers.

In 2004, Macfarlane et al.,^5^in a case-control study, suggested age and sex-specific STE cut-offs, applying analogous statistical techniques used in previous studies. However, the primary endpoint for diagnostic confirmation was again based on CK-MB values, ignoring the angiographic evidence of ACO.^5^Drawing from these case-control studies, the American Heart Association/American College of Cardiology Foundation/Heart Rhythm Society (AHA/ACCF/HRS) periodically redefines what is now known as the "STEMI criteria". This definition continues to be echoed in subsequent guidelines, including the 4th Universal Definition of MI.^6^

Shortly after the 2000 consensus was reached, doubts arose about the adequacy of the STEMI-NSTEMI paradigm. The label "STEMI" has inadvertently emphasized just one aspect of a diagnostic test— the presence of STE on the ECG.^7 – 9^Some physicians, possibly unaware of how the STEMI/NSTEMI paradigm originated, may mistakenly believe that patients with OMI, but lacking STE on their ECG, do not benefit from reperfusion therapy.

In 2001, Schmitt et al.^10^was among the first to study the STEMI-NSTEMI paradigm angiographically. They found that 29% of the 418 patients with ACO did not meet "STEMI criteria". In particular, 50% of ECGs of patients with acute left circumflex artery occlusion failed to meet criteria.^10^

In a post-hoc analysis of the PARAGON-B trial, 528 (27%) of 1,957 patients diagnosed with non-ST elevated acute coronary syndromes (NSTE-ACS) had fully occluded culprit vessels; these patients had larger infarct size and higher six-month risk-adjusted mortality.^11^

The TRITON-TIMI-38 study^12^provided additional evidence: of 1,198 patients with NSTE-ACS and isolated ST-segment depressions (STD), 314 (26.2%) had culprit arteries that were fully occluded. Koyama et al.^13^also contributed to this understanding, finding that in STEMI cases, 57% had TIMI-0 flow, while in NSTEMI cases, 47% had TIMI-0 flow, with both groups showing a mortality rate of around 5%.^13^This is evidence that NSTEMI with occluded coronary arteries are essentially equivalent to STEMI in terms of severity and outcomes.

In 2021, Meyers et al.^14^compared the STEMI/NSTEMI paradigm to the OMI/NOMI framework. They aimed to identify differences in catheterization timing and related outcomes between STEMI(+) OMI and STEMI(-) OMI groups. The authors found that 28% of NSTEMI patients had total coronary occlusion detected at delayed catheterization and 45% of OMI did not meet STEMI criteria. Infarct size of these STEMI (-) OMI were statistically equal to those of the STEMI(+) OMI group.^14^In further research, Meyers et al.^15^examined the accuracy of specific OMI ECG markers compared to the current STEMI criteria. These predefined OMI ECG markers were far more sensitive while maintaining high specificity. These data suggest that accurate ECG interpretation can quickly and noninvasively identify patients with STEMI(-) OMI for immediate reperfusion.^15^

The 2020 DIFOCCULT study^16^provided still more compelling evidence. Cardiologists, using expert interpretation of the ECG rather than strict STEMI criteria, blindly reclassified 28% of patients with NSTEMI as OMI; these patients had significantly higher long-term mortality than NSTEMI patients classified by ECG as having NOMI. A meta-analysis of diagnostic test accuracy conducted by our team further elucidates the diagnostic challenges associated with this condition. We found that the pooled sensitivity of STE in detecting ACO was only 43.6% (95% CI: 34.7%-52.9%), suggesting that more than half of ACO cases may not have STE. Specificity was high, at 96.5% (95% CI: 91.2%–98.7%). Further analysis employing the OMI-NOMI strategy demonstrated an improved sensitivity of 78.1% (95% confidence interval [CI]: 62.7%–88.3%) while maintaining a similar specificity of 94.4% (95% CI: 88.6%–97.3%).^17^

In a meta-analysis of prevalence, Khan et al.^18^reported that 25% of 40,000 patients with NSTEMI were found to have an acutely occluded artery on next day angiogram without any collateral circulation. Compared to NSTEMI patients with an open artery, these individuals faced nearly double the mortality rate, even though they were, on average, 15 years younger and had fewer comorbidities.^18^In another meta-analysis by Hung et al.,^19^of 60,000 NSTEMI patients, 34% were later found to have total coronary occlusion. Compared to patients with an open artery, those with OMI had, adjusted for other clinical factors: lower ejection fraction, higher biomarkers, more cardiogenic shock, and higher mortality (OR 1.72, 95% CI 1.49-1.98, p < 0.001).^19^

The STEMI-NSTEMI paradigm, by defining a disease by one very inaccurate aspect (STE) of one test (the ECG), overlooks the actual pathophysiology of ACO. This results in the "No False Negative Paradox:" if a patient has ACO but no STE, there is no STEMI and so there is no false negative test for STEMI. This has resulted in the exclusion of STEMI (-) OMI patients from STEMI databases, and thus in limited standardized data on the sensitivity and specificity of STE for diagnosing OMI. This data gap in the literature hinders clinical decision-making, as physicians must use potentially inadequate criteria to address acute coronary syndrome (ACS).

### Urgency of Treatment in Occluded Coronary Arteries: Time is Myocardium

In ACS, the saying "time is myocardium" holds true for OMI patients. For them, waiting is not an option; immediate reperfusion therapy is imperative. Troponin levels, while commonly used to diagnose MI, are not effective in diagnosing OMI in the acute settings for the following reasons:

- Time delay: Troponin results are not instantaneous; they take time both to acquire (blood draw) and to process, during which the myocardium is at risk;
- Lack of sensitivity: the initial high-sensitivity (hs) troponin lacks adequate sensitivity for acute OMI; in one study, the initial hs-troponin I was less sensitive than the rule-in threshold (52 ng/L) for type 1 MI in 27% of STEMI.^20^In a second real world population, the initial troponin value was below the upper reference limit in 47% of STEMI.^21^Moreover, troponin does not distinguish myocardial injury from AMI, type I MI from type II MI, nor OMI from NOMI. Consequently, hs-troponin is an unreliable marker for immediate clinical decision-making.

Perhaps the most common objection to the fact that NSTEMI-OMI [STEMI (-) OMI)] require emergent reperfusion comes from randomized trials comparing immediate vs. delayed interventions in patients with NSTEMI, showing no benefit. However, these studies are widely misrepresented. The most notable of these trials is the TIMACS study.^22^In this study, the "early intervention" group had a median intervention time of 16 hours, too prolonged to offer any benefit to patients with OMI. Furthermore, TIMACS did not include patients with ongoing symptoms. However, all studies that defined early intervention as less than two hours and included patients with ongoing symptoms demonstrated the benefit of an early intervention.^22 – 29^

Furthermore, the TIMACS study and others are often mischaracterized as involving only NSTEMI patients. However, this and other similar studies also enrolled patients without elevated troponin, who therefore, by definition, had unstable angina (UA), not AMI. It is well known that elevated troponin is a powerful predictor of high risk in ACS. In the case of TIMACS, 22% of the 3031 patients were diagnosed with UA, not AMI. It is worth noting that patients with serial negative troponins, indicative of UA, have never been shown to benefit from emergent rather than delayed treatment. How could more rapid reperfusion lead to reduced infarct size, when there is no infarct at all? Consequently, the lack of a noticeable difference in the primary outcome is not surprising, since 22% of the study population had no plausible mechanism of benefit.

Although the "early" intervention group in TIMACS waited an average of 16 hours for catheterization, there was a notable trend towards benefit in the primary outcome, with a 2.1% absolute risk reduction in death, MI, or stroke (9.4% vs. 11.5%, RR 0.81, 95%CI 0.63-1.04). This was observed despite the longer wait time for the "early" intervention group and the inclusion of 22% of patients with UA. Furthermore, the subgroup with a GRACE score > 140 showed benefits in all outcomes.

A subsequent important trial, frequently cited by those who claim no advantage over earlier NSTEMI management, is the VERDICT trial.^29^Among the 2,147 patients enrolled, 80% were diagnosed with NSTEMI, while 20% had UA without AMI. Again, the inclusion of UA dilutes the results. Adhering to all guidelines, the study excluded patients experiencing ongoing pain. In VERDICT, the angiogram times for the early and delayed groups were 4.7 and 62 hours, respectively. Although 4.7 hours is a significant improvement from the 16-hour duration observed in the early group of TIMACS, it remains a considerable delay in ACO-MI treatment, a fact readily acknowledged in the context of "STEMI" management. The primary outcome, which considered the potential benefits of rapid angiogram for a cohort comprising asymptomatic NSTEMIs and asymptomatic UA patients with serial negative troponins, unsurprisingly did not produce a discernible advantage for the early group.

However, like the TIMACS trial,^22^the VERDICT^29^subgroup with a GRACE score greater than 140 showed significant benefits in the primary outcome. Further examination of the NSTEMI patient subgroup revealed a 4% absolute reduction in the primary composite outcome (death, AMI, and repeated admission for ischemia or heart failure). This difference was not statistically significant, as the study could not discern a 4% variation (28.8% vs 32.7%, RR 0.85, 95% CI 0.71-1.01). An intervention demonstrating a reduction in absolute risk of 4% (NNT=25) in such a patient-centered outcome would hold significant clinical relevance if validated by a sufficiently powered study. So, the VERDICT findings cannot definitively counter this proposition.

Thus, the most extensive and pertinent studies^22 , 29^consistently indicate significant benefits in high-risk NSTEMI subgroups. These studies also suggest that the benefits observed in the entire NSTEMI subgroup could be confirmed if they were sufficiently powered. These findings come from populations where patients reported to be asymptomatic. Furthermore, the intervention times in the early management groups, 16 hours^22^and 4.7 hours^29^were substantially longer than what is typically considered emergent management (defined as less than 90 minutes). In fact, another trial, the RIDDLE-NSTEMI, where the time to percutaneous coronary intervention (PCI) was genuinely early (1.4 hours), demonstrated that an immediate invasive strategy in NSTEMI patients is associated with lower rates of death or new MI compared with a delayed invasive strategy.^30^This discrepancy hints at the potential for even more benefits if the intervention had been genuinely emergent, and especially if the study had been limited to OMI patients who are NSTEMI with specific ECG findings and usually with ongoing symptoms (e.g., persistent chest pain). Thus, our position is not to advocate for the emergent management of all NSTEMIs, especially those with resolved symptoms. Instead, we emphasize the importance of recognizing and promptly addressing the highest risk NSTEMI subgroup: OMI patients. These individuals, who have acutely occluded or nearly occluded culprit vessels, can gain the most from emergent management. The vast majority can be identified by the ECG using features beyond ST Elevation.

Given the critical anatomical reality of an occluded artery, it is recommended that the reperfusion therapy timeframe for OMI patients align with the door-to-needle and door-to-balloon time standards set for STEMI patients. Any delay in pursuing diagnostic clarity can lead to irreversible myocardial damage and negatively impact outcomes.

### The new paradigm: OMI-NOMI

Given the limitations inherent in the STEMI-NSTEMI paradigm, a more nuanced approach has been proposed, focusing on the anatomical and physiological intricacies of ACS. This revised paradigm distinguishes patients into two categories:

- OMI: This designation applies to patients experiencing ACO or near-occlusion, with limited collateral circulation, placing them at immediate risk of transmural AMI. This condition predominantly causes type 1 MI. The need for reperfusion therapy for these patients is urgent, regardless of the ECG findings. The primary concern is the anatomical obstruction and its associated risk, rather than the specifics of the ECG criteria. Although ECG findings play a role, other clinical and diagnostic findings, such as echocardiographic wall motion abnormalities or vessel obstruction detected by computed tomography coronary angiogram, can aid in diagnosing OMI, especially if the ECG is inconclusive.
- NOMI: This category includes patients without coronary occlusion. However, because NOMI may involve unstable ruptured plaque, they remain vulnerable to potential coronary thrombosis and ischemia. These patients may have elevated troponin levels or ECG changes, such as T-wave inversions. Although these patients need an angiogram and intervention if a culprit is found, immediate reperfusion therapy is not typically warranted for this group. ECG is not sensitive for NOMI but, fortunately, it need not be because immediate intervention is not needed, and the diagnosis can wait for troponin.

This revised framework seeks to enhance our comprehension and handling of ACS. It prompts clinicians to emphasize not just the ECG, but also the underlying pathophysiology, thus aligning treatments more closely with individual patient requirements (Central figure). Meyers and Smith first championed this paradigm in their "OMI Manifesto", which presents a significant direction for impending research and clinical application.

Advancements in technology have significantly influenced the refinement of AMI management. Emerging artificial intelligence algorithms exhibit high precision in diagnosing OMI and are rapidly evolving. Such advancements could enhance the OMI-NOMI paradigm, providing clinicians with even more precise diagnostic tools.^31 , 32^

### Other electrocardiographic signs of OMI

In the evolving landscape of OMI diagnosis, one of the key electrocardiographic signs is the hyperacute T-wave, characterized by increased area under the T wave relative to the QRS complex, including a broad base, increased length convexity, and a tendency towards symmetry. Visually, it appears as if the T-wave expands in all directions—towards the QRS complex, away from it, and upwards—resulting in a broadening of the QT interval and a more rounded peak ( Figure 1 ).

**Figure 1:** Hyperacute T waves; electrocardiogram at 25 mm/s showing a hyperacute T-wave in leads V2-V4.

Inverted T-waves are not indicative of hyperacute phases of infarction, as some might assume from the term "sub-epicardial ischemia". Inverted T-waves recorded by leads overlying the ischemic myocardium appear after acute reperfusion, and in sub-acute or chronic ischemic states, or as reciprocal to hyperacute T-waves in an opposing lead. When negative T-waves are present, the issue may not be located in the sub-epicardial zone of the overlying leads, but may be located in zones of distant, opposite myocardium.

In addition to these signs, the De Winter pattern is noteworthy. This pattern is estimated to occur in only about 2% of patients with anteroapical MI undergoing primary angioplasty. In the clinical context of acute ischemic chest pain, the De Winter ECG pattern exhibits a STD measured at the J point of at least 1 mm in leads V1-V2 to V6 followed by an usually upslope ST segment and a high, symmetrical, and positive hyperacute T wave ( Figure 2 ). Although de Winter T-waves receive a lot of attention, they have no more importance than other hyperacute T-wave, of which they comprise only a small fraction.

**Figure 2:** Subtle De Winter sign; electrocardiogram at 25 mm/s displaying the De Winter pattern, characterized by ST-segment depression at the J point in leads V1-V2 to V6, followed by an upsloping ST segment and a high, symmetrical, positive hyperacute T wave.

Transitioning from T-waves, terminal QRS distortion offers another diagnostic clue. Defined as the absence of an S-wave below the TP isoelectric line and the absence of a J-wave in leads V2 or V3, it is very specific finding for left anterior descending artery (LAD) when compared to early repolarization.^33^To differentiate physiological and ischemic STE in V2 and V3, the four-variable Smith's formula might be used, with 88.8% sensitivity and 94.7% specificity ( Figure 3 ).^34 , 35^The calculator is available in: Subtle Anterior STEMI Calculator (4-Variable) (mdcalc.com)

**Figure 3:** Subtle ST segment elevation in anteroapical wall; electrocardiogram displaying a subtle STE of approximately 1 mm in V1-V4, which does not fulfil the criteria set by the Universal Definition of Myocardial Infarction. The use of the 4-variable calculator indicated Occlusion Myocardial Infarction (OMI). Subsequent coronary angiography confirmed occlusion of the left anterior descending artery.

Another electrocardiographic sign of OMI is the Aslanger pattern, which is associated with inferior wall infarction and multi-vessel coronary artery disease (CAD), not meeting the classical criteria for STE in two contiguous leads. It is characterized by STE in lead III only, and STD in any of the leads V4-V6 but not in V2, and an ST segment in V1 that is greater than in V2.^36^

Another ECG sign of OMI involves occlusion of the diagonal branch, affecting the anteromedial wall of the heart. According to the current terminology for MI walls, which is based on magnetic resonance, leads I, aVL, and V2—and occasionally V3—correspond to the anteromedial wall of the left ventricle. In some cases, STE may only be evident in aVL and V2.^37 , 38^This region is supplied by the first diagonal branch of the LAD artery. A lack of awareness of this terminology could mislead cardiologists into thinking that aVL and V2 are not contiguous leads. When these leads are affected, a reciprocal change often occurs in the inferior wall, particularly in lead III. This specific pattern has been termed the "South African Flag" pattern.

The inverse also appears to be true: any ST depression in aVL is helpful to diagnose inferior wall OMI versus pericarditis. A study involving 426 patients with inferior MI and normal QRS complex found that 99% of ischemic inferior STEs exhibited some degree of reciprocal STD in lead aVL. This was true even when the inferior STE was subtle (less than 1 mm) and when there was STE in leads V5 and V6. In contrast, pericarditis showed no STD in any lead except aVR.^39^Another important change to consider is STD in leads V1 to V3/V4, which serves as a reciprocal indicator of STE in leads V7 to V9, corresponding to the lateral wall of the heart^31 , 40 , 41^

The diagnosis of AMI becomes particularly challenging when a patient presents with left bundle branch block (LBBB).^42^The 2013 ACC/AHA guidelines and the 2017^43^and 2023 ESC STEMI guidelines^44^recommend that patients with clinical suspicion of ongoing myocardial ischemia and LBBB should be managed irrespective of whether the LBBB is previously known. Importantly, the guidelines emphasize that the presence of a new or presumably new LBBB does not, by itself, predict an MI.^43 – 45^Sgarbossa et al.^46^proposed a scoring system that was further refined by introducing a proportional criterion, known as the Smith-modified Sgarbossa criteria, which is also validated for ventricular paced rhythm.^47 , 48^

According to the Fourth Universal Definition of MI,^6^new or presumably new RBBB without associated ST-segment or T-wave changes should be considered as a STEMI-equivalent ECG. These recommendations are primarily based on a retrospective study by Widimsky et al.,^49^which included 6,742 patients with acute MI.^49^In that study, not all participants underwent emergency angiography as a primary PCI protocol. Other evidences challenge the notion of RBBB as an indicator of emergency coronary angiography, as the likelihood of MI was similar to that of patients without any block.^50^Hence, further outcome data are needed for patients presenting with chest pain, presumably new RBBB, and no significant ST deviation.

To provide a succinct overview of the various signs and findings discussed, Figure 4 will summarize these elements for reference and better understanding.

**Figure 4:** Key electrocardiographic indicators for occlusion myocardial infarction.

### Current guidelines and their binary approach to acute MI diagnosis

The current guidelines for the management of AMI are meticulously crafted, serving as the foundation for treating of thousands of patients by cardiologists and general practitioners. These guidelines dictate that if a patient with OMI, but without ECG criteria for STEMI, arrives at the emergency department, it is unlikely that this patient will undergo urgent catheterization within the door-to-balloon time that is recommended for STEMI.^51^Instead, clinicians typically order a troponin test, which can take 1 to 2 hours to yield results. During this interval, patient often receive antianginal medications, including morphine. Although new point-of-care hs-troponins provide quick results,^52^an elevated troponin level confirms an AMI. If the clinical history is suggestive of type 1 MI, troponin still does not differentiate between OMI and NOMI. When the troponin level increases sufficiently to indicate OMI, substantial irreversible myocardial injury has already occurred, and the critical window for door-to-needle or door-to-balloon time has often closed. Thus, an OMI diagnosis should be determined before troponin results are available through expert ECG interpretation or application of artificial intelligence to the ECG. Morphine obscures the diagnosis by leading to a false sense of security, as the patient is apparently pain free (but not ischemia-free); thus, the use of morphine may be associated with prolonged times to angiography.^53^

The Brazilian guidelines for MI without STE recommend urgent invasive strategies for patients who experience refractory or recurrent chest pain.^33^This approach is designed to serve as a safety net for OMI patients who do not meet ECG criteria for STEMI. Catheterization is advised if a patient has a false-negative ECG but continues to experience chest pain. Although this recommendation is highly prudent, it is worth noting that its implementation is not as widespread as one might expect, even in settings where it is officially endorsed.^51^

In OMI, the initial troponin can either be positive or negative, similar to some obvious STEMI cases. Often, catheterization for NSTEMI is deferred to a later date. In more fortunate situations, it might take place later on the same day. However, both scenarios do not align with the timeframes recommended by current guidelines ( Figure 5 ).

**Figure 5:** Flowchart illustrating the current approach for acute myocardial infarction diagnosis; this has been the cornerstone for the treatment of numerous patients across various medical disciplines; the algorithm addresses the limitations of relying solely on electrocardiogram and troponin test results.

We appreciate the thoughtfulness and rigor in the development of these guidelines. However, we believe that there is an opportunity for more assertive action in this area. We advocate for the introduction of a new paradigm for approaching MI. This paradigm aims to be both more inclusive and accurate, providing a better framework for cardiologists, general practitioners, and medical students in the future.

### Our proposed approach for chest pain patients in the emergency department: the OMI-NOMI paradigm

In addressing the urgent need for a precise and rapid diagnosis of AMI, we propose a refined approach that emphasizes the differentiation between OMI and NOMI. If a patient with epidemiological risk factors presents to the emergency department with anginal pain (or other ischemic symptoms), the patient should be immediately evaluated for potential OMI ( Central Illustration ). This approach is designed to be both practical and effective, acknowledging the inherent complexities and limitations in diagnosing AMI.

Step-by-step diagnostic process:

1. Immediate ECG assessment: upon arrival at the emergency department, patients presenting with epidemiological risk factors and symptoms suggestive of ischemia (anginal pain or equivalents) should undergo an ECG within the first 10 minutes. This prompt evaluation is crucial in identifying potential cases of OMI.
2. Searching for STE in ECG: a key step in the evaluation of a patient with suspected AMI in the emergency department is the assessment of the STE on the ECG. While the presence of significant STE is a strong indicator of OMI, it is crucial to understand that approximately 30-50% of OMI cases may not exhibit this classic sign. This highlights the importance of not solely relying on STE for diagnosing OMI. Clinicians must be aware of this possibility and be prepared to investigate further, even in the absence of significant STE, to ensure that cases of OMI are not overlooked.
3. Looking for other signs of OMI: in addition to assessing for STE, clinicians should be vigilant in identifying other electrocardiographic indicators that may suggest OMI:^54 , 55^Subtle STE < 1 mm is frequently seen in acute OMI, and any STE of ≥ 1 mm in V2-V4 can be either normal or due to LAD artery OMI; use the four-variable formula to differentiate;^34^hyperacute T-waves with subtle STE or without any STE at all,^56^De Winter's sign,^57 , 58^Aslanger's pattern,^36 , 59 , 60^any STD in V1-V4 representing reciprocal changes from lateral wall (V7-V9),^41^terminal QRS distortion,^33^any STE in inferior leads accompanied by any STD in aVL indicative of reciprocal changes from the mid-anterior wall,^39^Smith's modified Sgarbossa criteria in instances of LBBB^47 , 61^or paced rhythm.^48^The presence of any of these signs necessitates immediate reperfusion. It is crucial to recognize that an OMI can occur even without these specific ECG signs, or even with a completely normal ECG.
4. Serial ECGs and comparison: In the absence of clear ECG signs of OMI, serial ECGs should be performed, and previous ECGs should be obtained for comparison. This step is vital in identifying dynamic changes that may indicate evolving myocardial ischemia.
5. Patients should receive antianginal treatment. Opioid pain relief should be avoided until the patient is committed to the cath lab, as it will hide ischemic symptoms. A patient with persistent chest pain in ACS requires an urgent invasive approach, even in the absence of ECG signs of OMI. Concurrently, it is essential to evaluate other potential causes of chest pain that may not respond to antianginal therapy ( Figure 6 ).

**Figure 6:** Proposed decision algorithm for managing chest pain in the emergency department. This flowchart outlines a comprehensive approach for the evaluation and management of patients presenting with chest pain in the emergency department. The algorithm incorporates both traditional and nuanced diagnostic criteria, aiming to improve the identification and treatment of Occlusion Myocardial Infarction (OMI). Key steps include initial clinical assessment, rapid electrocardiogram (ECG) evaluation, and consideration of both ST-segment elevation (STE) and other ECG signs. The algorithm also accounts for the possibility of false-negative OMI cases, emphasizing the importance of monitoring for refractory chest pain or dynamic changes in ECG as indicators for immediate reperfusion therapy. It is important to note that while this proposed algorithm offers a potential advancement in the management of chest pain, its implementation on a national scale, particularly within Brazil's Universal Healthcare System, may lead to an increase in the number of primary percutaneous coronary interventions. Therefore, the logistics and economic implications of this proposal should be carefully considered, and further validated through randomized controlled trials before widespread adoption.

Beyond ischemic symptoms, repeat ECGs, and troponin levels, various diagnostic tools can serve as supplementary indicators to identify OMI. Typically, echocardiography reveals a wall motion abnormality during an OMI, but bubble contrast, excellent technique, and experienced interpretation is necessary for high sensitivity. Finally, an emergency coronary CT angiogram should be easily accessible and analogous to the current approach to assessing acute stroke patients within the "large vessel occlusion" framework.^61^

While this approach aims to provide a clear and viable pathway for diagnosing AMI, we acknowledge that no diagnostic method is infallible. The complexity of AMI presentations means that there will always be cases where the diagnosis is not immediately clear. Our approach is designed to maximize diagnostic accuracy while being adaptable to the nuances of individual patient presentations.

## Conclusion

The AMI diagnosis landscape is poised for a paradigm shift. While the STEMI-NSTEMI framework has served us well as a transition out of the Reperfusion Era, mounting evidence and clinical observations indicate its limitations in addressing the intricate details of ACS. This article highlights these constraints and presents the OMI-NOMI paradigm as a more anatomically and physiologically accurate strategy for managing AMI. We anticipate that this refined approach will enhance patient outcomes by optimizing diagnostic accuracy and maximizing the efficacy of reperfusion therapies.

We have presented evidence challenging the diagnostic and therapeutic accuracy of the STEMI-NSTEMI paradigm, underscoring the notable rate of overlooked occlusion myocardial infarctions and the constraints of relying solely on the ECG (and especially on STE) and troponin levels for prompt clinical decisions. Additionally, we outline Instituto Dante Pazzanese's approach to diagnosing patients with chest pain in the emergency setting, stressing the importance of rapid evaluation of the ECG and immediate reperfusion therapy for potential cases of OMI.

We believe that the OMI-NOMI paradigm provides a promising direction for future research and clinical practice. It prompts clinicians to complement traditional diagnostic tests with a deeper understanding of the underlying pathophysiology of the ACS, leading to more tailored treatments to individual patient needs. We anticipate this article will stimulate further discussion and investigation within the Brazilian cardiology community, ultimately enhancing diagnostic precision and treatment efficacy for patients with AMI.
